# Abdominal type B vs. type C radical hysterectomy in early-stage cervical cancer: A matched single center cohort report

**DOI:** 10.3389/fsurg.2023.1166084

**Published:** 2023-04-12

**Authors:** Lu Wang, Ping Liu, Hui Duan, Pengfei Li, Guidong Su, Weili Li, Cong Liang, Chunlin Chen

**Affiliations:** Department of Obstetrics and Gynecology, Nanfang Hospital, Southern Medical University, Guangzhou, China

**Keywords:** cervical cancer, early-stage, type b radical hysterectomy, survival outcome, parametrectomy

## Abstract

**Objective:**

To compare survival outcomes of type B radical hysterectomy (RH) and type C RH in patients with early-stage cervical cancer.

**Methods:**

We retrospectively identified continuous cervical cancer patients with FIGO stage IA2-IB2 and IIA1 who underwent either type B RH (*n* = 278) or type C RH (*n* = 148) performed by the same group of surgeons between 2009 and 2018. Propensity score matching was carried out to minimize selection biases. Intraoperative photographs, immediate postoperative questionnaire and specimen measurements were used to accurately determine the extensive of surgery. We further narrowed the study population to patients with specific histological subtypes and patients with deep stromal invasion.

**Results:**

The median follow-up period was 42.41 ± 24.60 months. After adjusting, no differences in the 5-year overall survival (OS) and disease-free survival (DFS) were found between the type B group and the type C group (OS: 87.8% vs. 89.4%, *P *= 0.814; DFS: 84.9% vs. 85.6%, *P *= 0.898). In further analysis of patients with squamous-cell carcinoma, adenocarcinoma, adenosquamous carcinoma, similar 5-year OS and DFS rates were found between two groups (OS: 88.7% vs. 97.1%, *P *= 0.250; DFS: 84.7% vs. 92.3%, *P *= 0.541). Consistent results were found in patients with deep stromal invasion (OS: 81.8% vs. 100%, *P *= 0.144; DFS: 82.8% vs. 100%, *P *= 0.128).

**Conclusions:**

Type B RH could be used to treat FIGO stage IA2-IB2 and IIA1 cervical cancer to get equivalent 5-year OS and DFS. Further randomized controlled trials are warranted.

## Introduction

Despite the development of effective screening and vaccine, cervical cancer is still a major cause of cancer death in women worldwide. As clinical guidelines recommended, surgery is the primary treatment method for patients with early-stage disease, while concurrent chemoradiation therapy is the standard treatment approach in advanced cervical cancer. The latest National Comprehensive Cancer Network (NCCN) clinical practice guidelines for cervical cancer recommend radical hysterectomy and pelvic lymphadenectomy with an open abdominal approach as the standard surgical procedures for non-fertility-sparing early-stage cervical cancer patients. The recommendations for stage IA2 patients, and stage IB1, IB2 and selected IIB3-IIA1 patients who do not desire fertility preservation, are type B radical hysterectomy (RH) and type C RH, respectively (1, 2). For patients with advanced disease, it is still inconclusive whether salvage hysterectomy either extrafascial or radical is effective in persistent or recurrent disease after definitive chemoradiation therapy (3).

The main difference between type B and type C RH lies in the resection of parametrial tissues. Type C RH requires the resection of parametrial tissues at the junction with the internal iliac vascular system, while in type B RH, the transection is at the level of the ureter tunnel (4). Given that abundant vascular and nerve tissues are located around the cervix, removing all the parametrial tissues during type C RH would increase the risk of complications, such as bleeding, bladder and rectal injuries, and multiple dysfunctions due to the damage to the pelvic autonomic nerves, which severely distresses patients (5–10). In addition, several studies reported a relatively low risk (0%–10.8%) of parametrial infiltration (PI) in some patients with early-stage cervical cancer, and this risk decreases to 0% in patients with tumor sizes < 2 cm, infiltration depth < 10 mm, negative pelvic lymph nodes and absent LVSI (11–13). These findings suggest the possibility of narrowing the extent of parametrial resection or even omitting parametrectomy in selected low-risk early-stage cervical cancer patients, thus reducing the morbidity of surgery-related complications.

In recent years, some studies have demonstrated that type B RH is comparable to type C RH in terms of oncological outcome for selective patients with early-stage cervical cancer (14–17). A review in 2020 also summarized that reduced radicality on the parametrium offers positive effects on the quality of life (sexual life and bladder function) of patients without impacting on survival, oncological outcome (18). However, no final conclusion has been made, and some studies have not taken into consideration the impacts of pathological risk factors and adjuvant therapy on survival. In our previous research, we found that in the real world, if revised by the key step of parametrectomy in surgical records, the proportion of abdominal type B RH in early-stage cervical cancer patients is approximately 77.29%, while the proportion of type C RH is approximately 22.71%, and type B is not inferior to type C RH in terms of oncological outcome (19). However, the previous study is a retrospective study although it included in multi-center data. The definition of the surgery type mainly relied on the original surgical record and lack of objective verification, so there was some uncertainty.

Our aim was to evaluate the survival outcome of abdominal type B and type C RH by precisely defining the surgery types comprehensively based on data from the same group of surgeons in a single center, intraoperative photographs, an immediate postoperative questionnaire, specimen measurements, and propensity score matching.

## Methods

This retrospective cohort study was approved by the Ethics Committee of Nanfang Hospital, Southern Medical University (NFEC-2017-135) and was performed in accordance with the principles of the Declaration of Helsinki. The requirement for informed consent was waived.

### Study population

We retrospectively enrolled 426 consecutive cervical cancer cases from January 1, 2009, to April 30, 2018. The inclusion criteria for the study were as follows: (1) patients who were aged over 18 years; (2) patients who were diagnosed with stage IA2, IB1, IB2, and IIA1 cervical cancer; and (3) patients who underwent abdominal type B or C RH and pelvic lymphadenectomy with or without para-aortic lymphadenectomy. The exclusion criteria were as follows: (1) patients who did not undergo pelvic lymphadenectomy or whose lymph node resection type was unknown; and (2) patients who were diagnosed with cervical cancer during pregnancy, those with occult cervical cancer found after simple hysterectomy, and those with synchronous secondary malignant tumors. Additional inclusion criteria for subgroup analysis were as follows: (1) patients whose histological types were squamous cell carcinoma, adenocarcinoma or adenosquamous carcinoma; and (2) patients who did not receive neoadjuvant chemotherapy or radiology prior to surgery. Patients with deep stromal invasion underwent further analysis.

### Data collection

According to our previously published articles, data on 315 parameters were collected by well-trained gyneacologists from medical documents (19–21). Clinical stage was revised in accordance with the FIGO 2018 standards (22). Adjuvant therapy was delivered based on postoperative pathological risk factors. Patients who had either one high risk factor (parametrial invasion, positive vaginal resection margin, lymph node metastasis) received postoperative chemoradiotherapy, and those who had any two intermediate risk factors (tumor size more than 4 cm, lymphovascular invasion, deep stromal invasion) required radiotherapy (23).

### Definition of surgery type

All surgeries in this study were performed by four doctors of the same group. The type of surgery was defined according to Querleu and Morrow's classification (4). For type B RH, the uterine artery and cardinal ligament were resected at the level of the ureteral tunnel, and the uterosacral ligament was partially removed. Type C RH requires transection of the uterine artery and the cardinal ligament at the iliac vessels, and uterosacral ligament at the rectum. All surgical procedures were photographed. Questionnaires to record the exact resection range of the uterine vessels, the cardinal ligament and the uterosacral ligament were completed by the surgeons immediately after the surgery. Postoperative specimens with scale plates were photographed and the lengths of the cardinal ligament, uterosacral ligament and vagina were recorded for later verification and analysis. The definition of surgery type was mainly based on the questionnaires, but were amended when discrepancies were found between questionnaires and image documents including intraoperative photographs and postoperative measurement. All the data were archived.

### Propensity score matching

To eliminate differences in the baseline characteristics and reduce the possible selection bias, we selectively included factors such as age, FIGO stage, histological type, LVSI status, depth of stromal invasion, lymph node metastasis, vaginal margin, and parametrial invasion to perform 1: 2 propensity score matching.

### Observation indicators

The main outcomes were the overall survival (OS) and disease-free survival (DFS) of patients in the type B and type C RH groups. The follow-up frequency was recommended every 3 to 6 months for 2 years, every 6 to 12 months for another 3 to 5 years, and then annually. The endpoint of observation was the long-term survival outcome at 5 years. We defined OS as the time interval between the date of surgery and either the date of death due to any cause or the date of the last valid follow-up. DFS was defined as the time interval between the date of surgery and either the date of disease relapse/death or the date of the last valid follow-up. Recurrence was diagnosed when new-onset symptoms and mass of the pelvic, abdominal or pulmonary occur as confirmed by biopsy or imaging scans.

### Statistical analysis

SPSS version 26.0 (IBM Corporation, Armonk, NY, USA) was used for statistical analysis. Comparisons were performed *via* Student's t test for continuous variables and Pearson's chi-squared test or Fisher's exact test for categorical variables. Survival analysis was performed using the Kaplan-Meier method, log-rank test and Cox proportional hazards regression model to compare the OS and DFS for 5 years with hazard ratios (HRs) and 95% confidence intervals (95% CIs). The difference was considered statistically significant when *P *< 0.05.

## Results

**Figure 1:**
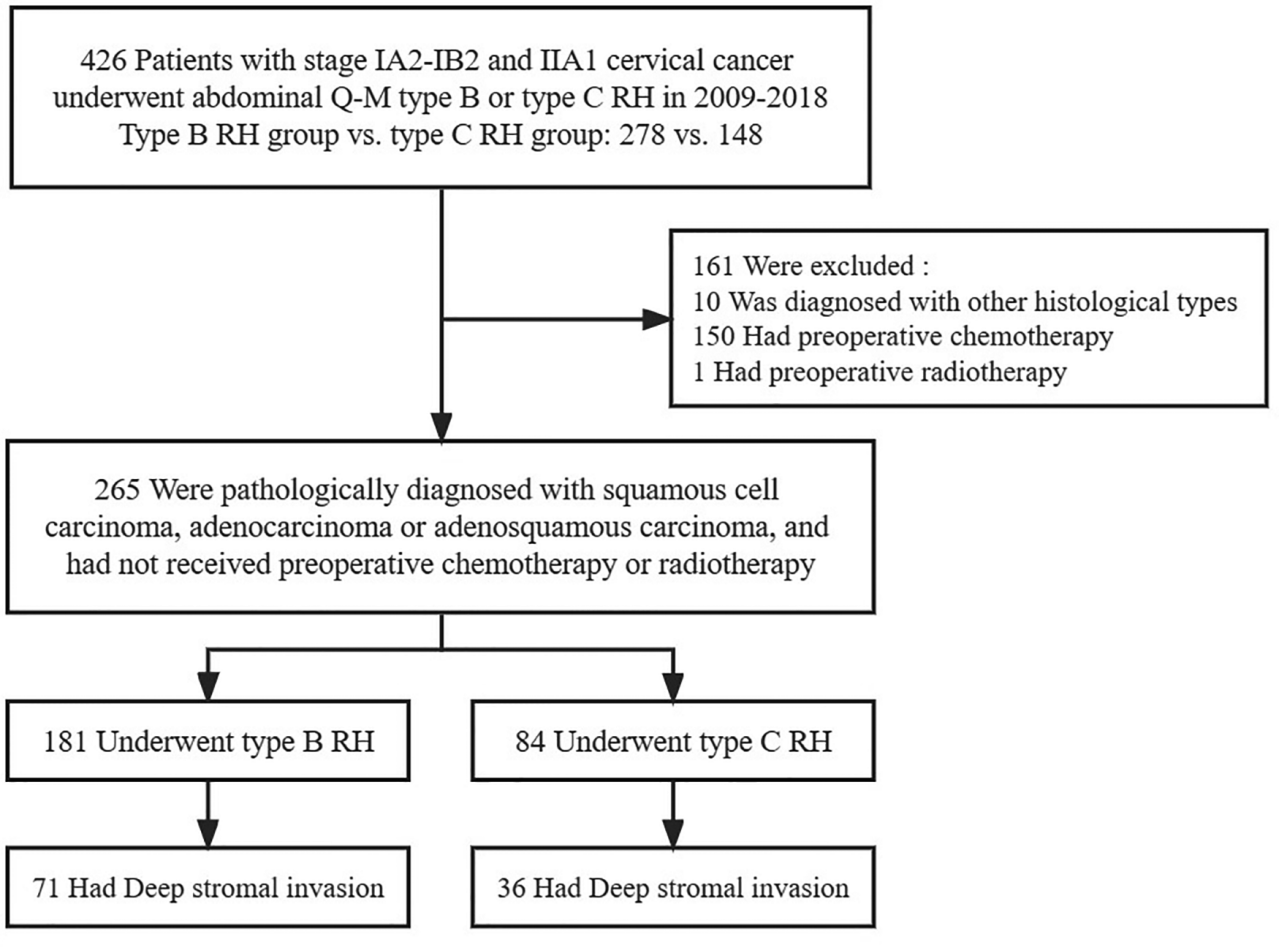
Study population.

The selection of the study population is shown in Figure 1. In total, 426 patients with FIGO stage IA2-IIA2 cervical cancer were included in this study. The basic characteristics of all the patients are depicted in Table 1. All cases were included for the initial analysis, including 278 patients in the type B RH group and 148 in the type C RH group. Then, 265 patients with squamous cell carcinoma, adenocarcinoma, and adenosquamous carcinoma were included in the further analysis, of whom 181 underwent type B RH and 84 underwent type C RH. Futhermore, 107 patients with deep stromal invasion were included for additional analysis.

**Table 1:** Clinicopathological characteristics of inclusive early-stage cervical cancer patients (*n* = 426).

Variables	Values (percentage)
Age, y	49.21 ± 9.17 (25–71)
Follow-up time	42.41 ± 24.60 (0–112)
Length of resected CL, cm	2.48 ± 0.92 (1.20–4.50)
Length of resected USL, cm	2.77 ± 1.02 (1.30–5.80)
Histological subtype	
Squamous-cell carcinoma	374 (87.79)
Adenocarcinoma	38 (8.92)
Adenosquamous carcinoma	4 (0.94)
Other subtypes	10 (2.35)
FIGO stage	
IA2	35 (8.22)
IB1	156 (36.62)
IB2	103 (24.18)
IIA1	132 (30.98)
Depth of tumor invasion	
≤ 1/2	196 (46.01)
> 1/2	183 (42.96)
Unknown	47 (11.03)
LVSI	
Negative	364 (85.45)
Positive	62 (14.55)
Parametrial involvement	
Negative	413 (96.95)
Positive	13 (3.05)
Vaginal margin	
Negative	343 (80.52)
Positive	83 (19.48)
Lymph node involvement	
Negative	341 (80.05)
Positive	85 (19.95)

### Survival analysis of patients with FIGO stage IA2-IB2 and IIA1 cervical cancer

**Figure 2:**
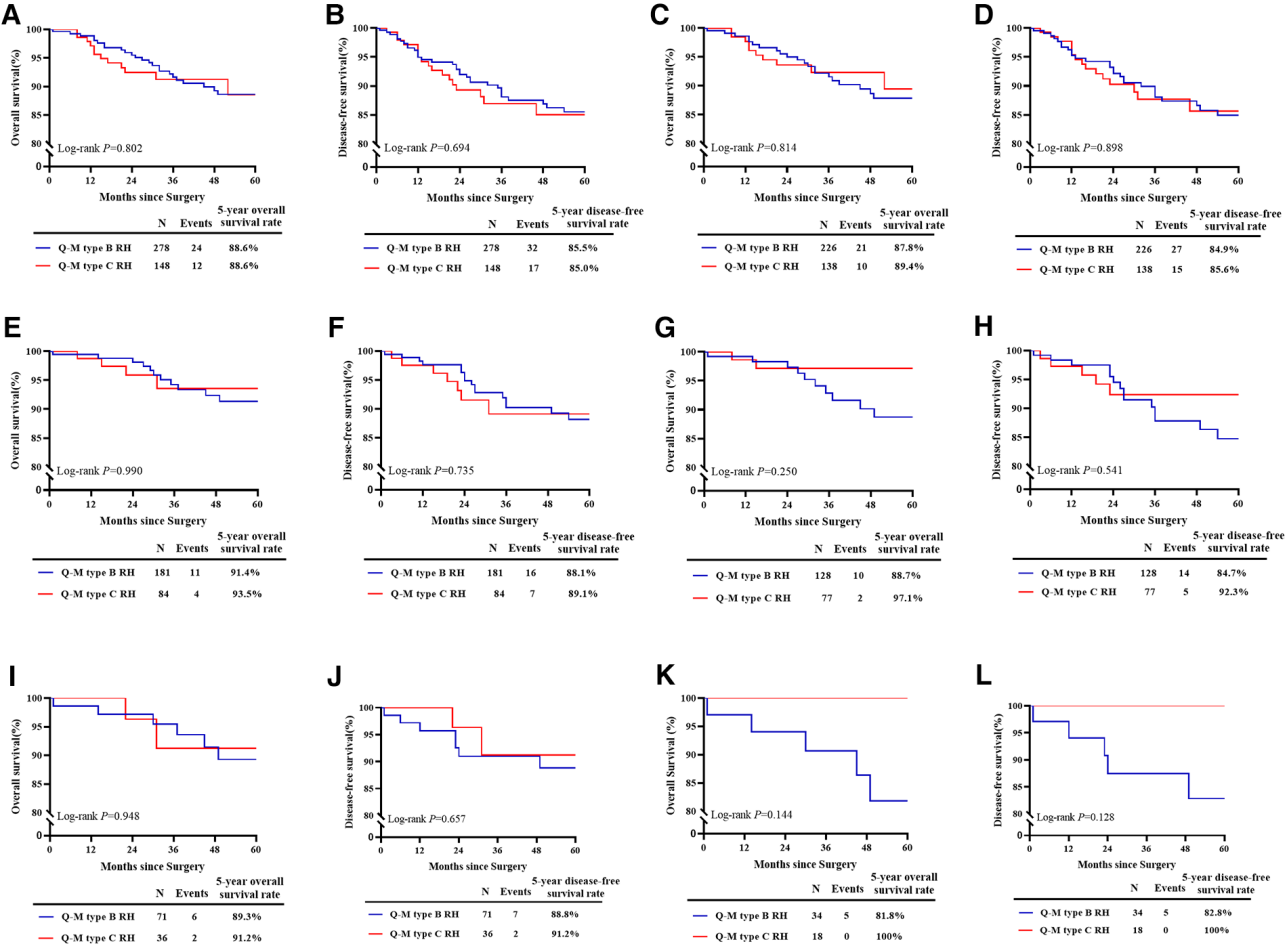
Survival estimates of patients with FIGO stage IA2-IB2 and IIA1 cervical cancer. (**A**): The 5-year OS of all patients in the real world (*n* = 426). (**B**): The 5-year DFS of all patients in the real world (*n* = 426). (**C**): The 5-year OS after matching (*n* = 364). (**D**): The 5-year DFS after matching (*n* = 364). (**E**): The 5-year OS of patients with specific histological subtypes (*n* = 265). (**F**): The 5-year DFS of patients with specific histological subtypes (*n* = 265). (**G**): The 5-year OS of patients with specific histological subtypes after matching (*n* = 205). (**H**): The 5-year DFS of patients with specific histological subtypes after matching (*n* = 205). (**I**): The 5-year OS of patients with deep stromal invasion (*n* = 107). (**J**): The 5-year DFS of patients with deep stromal invasion (*n* = 107). (**K**): The 5-year OS of patients with deep stromal invasion after matching (*n* = 52). (**L**): The 5-year DFS of patients with deep stromal invasion after matching (*n* = 52).

The clinicopathological characteristics of the patients are presented in Table 2. Baseline analysis between the type B and type C RH groups revealed differences in histological type (*P *= 0.041), PI (1.08% vs. 6.76%, *P *= 0.001), and positive vaginal margin (15.83% vs. 26.35%, *P *= 0.009). The median follow-up period was 42.41 months, during which 49 patients (11.50%) experienced recurrence and 36 patients (8.45%) died. There were no differences in 5-year OS or DFS between the type B and type C RH groups (5-year OS: 88.6% vs. 88.6%, *P *= 0.802; 5-year DFS: 85.5% vs. 85.0%, *P *= 0.694) (Figures 2A, B). Then we performed 1: 2 propensity score matching, and 226 patients in type B group and 138 in type C group were included. There were no differences in 5-year OS or DFS between the two groups (OS: 87.8% vs. 89.4%, *P *= 0.814; DFS: 84.9% vs. 85.6%, *P *= 0.898) (Figures 2C, D). Cox multivariate analysis showed that the type of RH was not an independent risk factor for 5-year OS and DFS (Table 3).

**Table 2:** Characteristics of early-stage cervical cancer patients, before and after 1:2 propensity score matching.

	All patients			Patients with specific pathological subtypes			Patients with deep stromal invasion		
	Type B RH group (*n* = 278,%)	Type C RH group (*n* = 148,%)	*P* Value	Type B RH group (*n* = 181,%)	Type C RH group (*n* = 84,%)	*P* Value	Type B RH group (*n* = 71,%)	Type C RH group (*n* = 36,%)	*P* Value
Age, y	49.12 ± 9.17	49.40 ± 9.21	0.762	49.48 ± 9.29	50.57 ± 9.04	0.364	49.23 ± 9.43	51.83 ± 10.02	0.189
Length of resected CL, cm	2.25 ± 0.71	2.88 ± 1.11	<0.001	2.25 ± 0.73	2.99 ± 1.07	<0.001	2.29 ± 0.62	3.15 ± 1.04	<0.001
Length of resected USL, cm	2.60 ± 0.91	3.08 ± 1.13	<0.001	2.57 ± 0.88	3.16 ± 1.28	<0.001	2.58 ± 0.96	3.23 ± 1.37	0.001
Histological subtype			0.041			0.080			0.355
Squamous-cell carcinoma	242 (87.05)	132 (89.19)		165 (91.16)	72 (85.71)		66 (92.96)	32 (88.89)	
Adenocarcinoma	25 (8.99)	13 (8.78)		16 (8.84)	10 (11.90)		5 (7.04)	3 (8.33)	
Adenosquamous carcinoma	1 (0.36)	3 (2.03)		0 (0)	2 (2.38)		0 (0)	1 (2.78)	
Other subtypes	10 (3.60)	0 (0)		–	–		–	–	
FIGO stage			0.103			0.075			0.717
IA2	22 (7.91)	13 (8.78)		18 (9.94)	5 (5.95)		5 (7.04)	2 (5.56)	
IB1	102 (36.69)	41 (27.70)		82 (45.30)	28 (33.33)		23 (32.39)	15 (41.67)	
IB2	70 (25.18)	33 (22.30)		40 (22.10)	21 (45.00)		31 (43.66)	12 (33.33)	
IIA1	84 (30.22)	61 (41.22)		41 (22.65)	30 (35.71)		12 (16.90)	7 (19.44)	
Depth of tumor invasion			0.190			0.612	–	–	–
≤ 1/2	135 (48.56)	61 (41.22)		93 (51.38)	38 (45.24)		–	–	–
> 1/2	117 (42.09)	66 (44.59)		71 (39.23)	36 (42.86)		–	–	–
Unknown	26 (9.35)	21 (14.19)		17 (9.39)	10 (11.90)		–	–	–
LVSI			0.307			0.906			0.875
Negative	234 (84.17)	130 (87.84)		154 (85.08)	71 (84.52)		60 (84.51)	30 (83.33)	
Positive	44 (15.83)	18 (12.16)		27 (14.92)	13 (15.48)		11 (15.49)	6 (16.67)	
Parametrial involvement			0.001			0.006			0.001
Negative	275 (98.92)	138 (93.24)		180 (99.45)	79 (94.05)		71 (100)	31 (86.11)	
Positive	3 (1.08)	10 (6.76)		1 (0.55)	5 (5.95)		0 (0)	5 (13.89)	
Vaginal margin			0.009			0.014			0.016
Negative	234 (84.17)	109 (73.65)		156 (86.19)	62 (73.81)		60 (84.51)	23 (63.89)	
Positive	44 (15.83)	39 (26.35)		25 (13.81)	22 (26.19)		11 (15.49)	13 (36.11)	
Lymph node involvement			0.164			0.582			0.713
Negative	228 (82.01)	113 (76.35)		148 (81.77)	71 (84.52)		55 (77.46)	29 (80.56)	
Positive	50 (17.99)	35 (23.65)		33 (18.23)	13 (15.48)		16 (22.54)	7 (19.44)	

**Table 3:** Cox regression of the included cervical cancer patients.

	All patients		Patients with specific pathological subtypes		Patients with deep stromal invasion	
	OS	DFS	OS	DFS	OS	DFS
	*P* Value (HR, 95% CI)	*P* Value (HR, 95% CI)	*P* Value (HR, 95% CI)	*P* Value (HR, 95% CI)	*P* Value (HR, 95% CI)	*P* Value (HR, 95% CI)
Type of RH	0.694 (0.866, 0.423 to 1.773)	0.733 (0.897, 0.481 to 1.673)	0.809 (0.856, 0.241 to 3.033)	0.746 (0.846, 0.307 to 2.330)	0.357 (0.302, 0.024 to 3.852)	0.302 (0.292, 0.028 to 3.021)
Histological subtype	0.373 (1.275, 0.747 to 2.177)	0.004 (1.578, 1.154 to 2.157)	0.112 (3.362, 0.755 to 14.974)	0.020 (3.484, 1.222 to 9.933)	0.069 (4.299, 0.891 to 20.739)	0.108 (3.462, 0.761 to 15.746)
FIGO stage	0.015 (1.493, 1.080 to 2.064)	0.026 (1.351, 1.036 to 1.761)	0.374 (1.271, 0.749 to 2.156)	0.296 (1.246, 0.825 to 1.881)	0.898 (1.052, 0.485 to 2.282)	0.845 (0.931, 0.454 to 1.910)
Depth of stromal invasion	0.291 (0.750, 0.440 to 1.279)	0.361 (0.813, 0.521 to 1.268)	0.945 (1.033, 0.403 to 2.648)	0.597 (0.831, 0.471 to 1.655)	–	–
LVSI	0.097 (1.918, 0.889 to 4.139)	0.269 (1.483, 0.738 to 2.982)	0.425 (1.727, 0.452 to 6.607)	0.833 (0.880, 0.268 to 2.893)	0.859 (0.803, 0.072 to 8.978)	0.813 (0.755, 0.074 to 7.740)
Vaginal margin	0.807 (1.095, 0.527 to 2.275)	0.690 (1.140, 0.598 to 2.173)	0.310 (1.961, 0.535 to 7.195)	0.086 (2.505, 0.879 to 7.141)	0.100 (4.040, 0.767 to 21.280)	0.119 (3.562, 0.720 to 17.613)
Parametrial invasion	0.330 (1.858, 0.534 to 6.463)	0.641 (1.336, 0.396 to 4.052)	0.603 (1.857, 0.180 to 19.128)	0.890 (1.165, 0.133 to 10.168)	0.255 (6.516, 0.258 to 164.699)	0.327 (4.729, 0.212 to 105.506)
Lymph node involvement	0.001 (5.526, 0.2.657 to 11.491)	0.001 (4.410, 2.359 to 8.247)	0.097 (2.780, 0.831 to 9.300)	0.038 (2.810, 1.061 to 7.441)	0.676 (1.488, 0.231 to 9.570)	0.895 (1.125, 0.193 to 6.546)

### Further survival analysis of patients with specific pathologic features

For patients with specific pathological subtypes, differences were found in PI (0.55% vs. 5.95%, *P *= 0.006), and positive vaginal margin (13.81% vs. 26.19%, *P *= 0.014), as shown in Table 2. The median follow-up period was 42.57 months, during which 23 patients (8.68%) experienced recurrence and 15 patients (5.66%) died. There were no differences in 5-year OS or DFS between the type B and type C RH groups before matching (5-year OS: 91.4% vs. 93.5%, *P *= 0.990; 5-year DFS: 88.1% vs. 89.1%, *P *= 0.735) (Figures 2E, F) and after matching (OS: 88.7% vs. 97.1%, *P *= 0.250; DFS: 84.7% vs. 92.3%, *P *= 0.541) (Figures 2G, H). For patients with deep stromal invasion, differences were found in PI (0% vs. 13.89%, *P *= 0.001), and positive vaginal margin (15.49% vs. 36.11%, *P *= 0.016), as shown in Table 2. The median follow-up period was 39.79 months, during which 9 patients (8.41%) experienced recurrence and 8 patients (7.48%) died. There were no differences in 5-year OS or DFS between groups before matching (5-year OS: 89.3% vs. 91.2%, *P *= 0.948; 5-year DFS: 88.8% vs. 91.2%, *P *= 0.657) (Figures 2I, J) and after matching (OS: 81.8% vs. 100%, *P *= 0.144; DFS: 82.8% vs. 100%, *P *= 0.128) (Figure 2K, L). Cox multivariate analysis showed that the type of RH was not an independent risk factor for 5-year OS and DFS (Table 3).

## Discussion

Despite the recommendation of surgery modality in international guidelines, the present trend of developing cervical cancer at a relatively young age has making the quality of life and physiological function a significant concern that cannot be ignored. From the anatomical point of view, more nerves within the CL are removed in regular type C RH (24). Reasonably narrowing down the resection range of the parametrial tissue and the upper vagina would decrease the morbidity of complications. To find the best candidates for tailoring parametrectomy in cervical cancer, researchers have conducted a series of studies.

Lower operative time and postoperative time, lower morbidity of fistula-related complications and lower recurrence rate of Piver type II RH compared to type III RH were found (25). However, they only included very early-stage occult cancer of the cervix that were diagnosed after conization for type II RH in this study, while patients who underwent type III RH had more advanced diseases. Ditto A et al. (17) retrospectively included patients with FIGO stage IA2, IB1, and IIA1 cervical cancer and found that patients in class III group had higher risk of death (HR = 3.08) and recurrence (HR = 2.51) than those in class II group as confirmed by Cox multivariable analysis. However, the patients included in this study were non-synchronous surgical patients and baseline differences in clinical stage, histological grade, LVSI, and vaginal margin were found between groups. Thus, to eliminate the baseline differences between groups, we performed 1: 2 propensity score matching to compare oncological outcomes in a more accurate way.

A Chinese cohort study (16) found similar OS and DFS between class II and class III radical hysterectomy in patients with tumor size no greater than 2 cm and grade 1 to 2 squamous cell cancer. It is not clear whether type II RH is applicable to patients of other stages. Panici PB et al. (26) included cervical cancer patients with stage IA2-IB1 disease and conducted type II RH for patients with negative lymph nodes and type III-IV RH for patients with positive lymph nodes through intraoperative frozen pathology. Higher 5-year OS was found in the type II RH group than type III-IV RH group (95% vs. 74%). Yet, selection bias was inevitable on account of the poor prognostic risk of lymph node metastasis. Similarly, Bezerra AL et al. (27) found that in stage I-IIA patients with squamous cell carcinoma or adenocarcinoma, type II RH achieved similar results as type III RH. However, 9 patients in this study received adjuvant therapy before surgery. Real-world evidence indicated that preoperative radiotherapy could reduce the incidence of deep cervical stromal invasion and LVSI (21). In this study, we included patients of stage IIA1 to further explore the possible candidates for type B RH and excluded patients that received preoperative chemotherapy and radiotherapy to avoid bias.

The shorter remained length of vagina and radiotherapy-related complications would also be risk factors of poor quality of life. The mid-term results of an RCT comparing the efficacy of Piver type II and type III RH (28) showed comparable 2-year DFS between groups, but further evaluation of long-term efficacy and safety is absent. Besides bladder function scores, the postoperative vaginal and sexual function scores of the type II RH group were all lower than type III RH group. Satisfactory postoperative symptoms were achieved. Landoni F et al. (29) conducted a prospective randomized study and expanded the indication of type II RH to stage IB-IIA cervical cancer. They identified cervical diameter as a significant predictor of the need for adjuvant radiotherapy because 80% (47/59) of patients with cervical diameter greater than 4 cm delivered to radiotherapy, which lead to additional morbidity. In the current study, around 20% patients from each group received adjuvant radiotherapy and no extra radiotherapy were conducted due to the narrowing of the surgery.

In addition, we found that 13 out of the 426 patients (3.05%) with stage IA2-IB2 and IIA1 cervical cancer had PI, suggesting the possibility that surgeons may reduce the extensive of parametrial tissue resection of early-stage cervical cancer. Researchers from Canada (30) have reported that the PI rate is approximately 4% (33/842) for patients with stage IA1-IB1 cervical cancer and 0.6% (3/356) for cervical cancer patients with a tumor size of 2 cm or less, negative pelvic lymph nodes and cervical stromal invasion less than 10 mm. Wright JD et al. (12) reported PI rate of cervical cancer was approximately 10.8% (64/594) and discovered poor differentiation, deep stromal invasion, LVSI, large tumor size, advanced stages, uterine or vaginal invasion, and lymph node metastasis as risk factors for PI. For patients with positive pelvic lymph node, PI rate was 47.9% (34/71), whereas PI rate was as low as 0.4% in patients who had negative lymph nodes, negative LVSI, and a tumor size of less than 2 cm. However, most of the previous studies that reported PI only included patients with stage IB or earlier cervical cancer. Our study broadened the clinical stage to stage IIA1, and we still found a low rate of PI and a content prognosis, which may shed light on the possibility of narrowing the surgical extensive.

This study has certain limitations. First, despite its prospective study design, it is a single-center, non-RCT study. Second, in this study, we did not compare the complications and quality of life and could not obtain a sufficient evaluation of surgery safety. In addition, we did not systematically analyze the patterns of PI, the positive rates of PI in different stages, or the risk factors of PI. However, we adopted propensity score matching to adjust bias as much as possible. Furthermore, for the definition of different RHs, we adopted the methods of taking photographs during surgery, filling out questionnaires immediately after surgery and measuring specimens. The operation technique of RH is a significant influence factor for survival, thus emphasis should be put on the admittance of the surgery. Referral to experienced gynecologic oncologists in tertiary hospitals should be considered if conditions permit. Further RCT to thoroughly compare the oncological outcomes and evaluate the safety of the two types of RH is undergoing (NCT04691453).

## Conclusions

Type B RH could be used to treat stage IA2-IB2 and IIA1 cervical cancer to get equivalent 5-year OS and DFS. Further randomized controlled trials are warranted.

## Data Availability

The raw data supporting the conclusions of this article will be made available by the authors, without undue reservation.
